# Comparative analysis of therapeutic strategies in atrial fibrillation patients with left atrial appendage thrombus despite optimal NOAC therapy

**DOI:** 10.1007/s00392-025-02665-w

**Published:** 2025-05-06

**Authors:** Ferenc Komlósi, Bence Arnóth, Imre Szakál, Patrik Tóth, Henriette Mészáros, Helga Sánta, Gyula Bohus, Péter Vámosi, Elektra Bartha, Márton Horváth, Melinda Boussoussou, Nándor Szegedi, Zoltán Salló, István Osztheimer, Péter Perge, Gábor Széplaki, László Gellér, Béla Merkely, Klaudia Vivien Nagy

**Affiliations:** 1 Heart and Vascular Center, Semmelweis University, Budapest, Hungary; 2 Faculty of Medicine, Semmelweis University, Budapest, Hungary; 3 Mater Private Hospital, Dublin, Ireland

**Keywords:** Atrial fibrillation, Left atrial appendage thrombus, Oral anticoagulation, NOAC therapy

## Abstract

**Background and aims:**

Left atrial appendage (LAA) thrombus is the primary cause of stroke and systemic embolism in atrial fibrillation (AF). Non-vitamin-K oral anticoagulants (NOACs) effectively reduce LAA thrombus prevalence and stroke risk. However, the optimal treatment of a NOAC-resistant thrombus remains unclear. We aimed to evaluate therapeutic strategies for resolving LAA thrombus in patients on optimal NOAC therapy.

**Methods:**

We retrospectively analyzed patients scheduled for cardioversion or catheter ablation of AF between 2014 and 2023 with LAA thrombus on transesophageal echocardiography (TEE) despite being on optimal NOAC therapy. We assessed how the applied management strategy affected thrombus resolution.

**Results:**

Among the analyzed 120 patients, a change to a different NOAC occurred in 41% of cases, a transition to a VKA in 30%, and the supplementation with antiplatelet therapy in 11%. In contrast, 18% of the patients received unchanged therapy. Follow-up imaging at 65 [44 – 95] days showed successful thrombus resolution in 92 (77%) of cases, predicted by a lower CHA2DS2-VASc score (*p* = 0.01). Any modification of antithrombotic therapy was an independent predictor of thrombus resolution (OR 5.28 [1.55–18], *p* = 0.01). Of the four strategies, there was a trend toward better thrombus resolution with switching to a VKA (OR 3.23 [1.03–10.1], *p* = 0.04).

**Conclusion:**

Resolution of LAA thrombus in patients already on adequate NOAC treatment may require a revision of the anticoagulation strategy. In addition, transitioning from NOAC to VKA might be considered.

**Graphical abstract:**

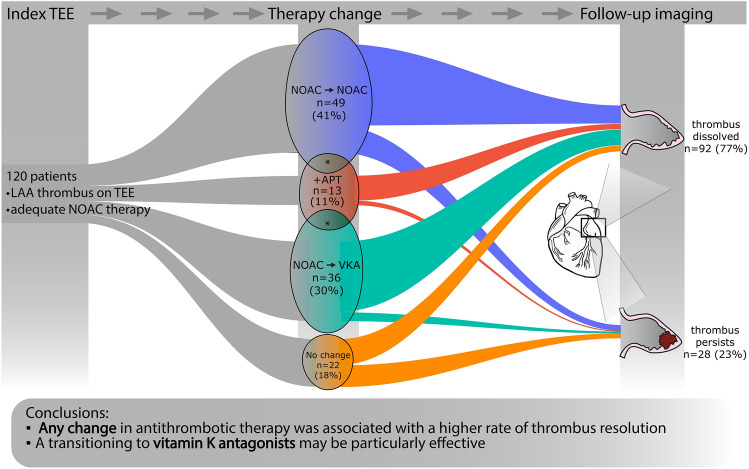

**Supplementary Information:**

The online version contains supplementary material available at 10.1007/s00392-025-02665-w.

## Introduction

Atrial fibrillation (AF) is the most common supraventricular arrhythmia and one of the leading causes of stroke and systemic thromboembolic events [1]. The left atrial appendage (LAA) is recognized as the primary site of thrombus formation [2]. Thus, an LAA thrombus is associated with an increased risk of thromboembolism and contraindicates any therapy to restore sinus rhythm [3]. Consequently, despite growing evidence supporting the benefits of rhythm control [4–9], these patients receive rate control until thrombus resolution is confirmed. Anticoagulant therapy with oral anticoagulants (OAC) is recommended to reduce stroke risk in AF patients based on the CHA2DS2-VASc clinical stroke risk score [8]. Both vitamin K antagonists (VKA) and novel oral anticoagulants (NOAC) are effective and safe in preventing thromboembolism [10]. According to the current guidelines, NOACs are preferred over VKAs due to their lower bleeding risk, and recent studies suggest that NOACs may offer superior stroke prevention [11].

Transesophageal echocardiography (TEE) is considered the gold standard modality in identifying LAA thrombus (LAAT) [12], while intracardiac ultrasound and cardiac computed tomography (CT)are widely used alternatives [13]. Imaging is recommended to exclude LAA thrombosis before electrical cardioversion (ECV) or catheter ablation (CA) in patients who have not received at least 3 weeks of OAC therapy. Although cerebrovascular complications are rare [14, 15], routine LAA thrombus exclusion is often performed before these procedures despite chronic anticoagulant treatment to further minimize thromboembolic risk [16].

Despite adherence to guideline-directed effective OAC dosing, LAA thrombi are found in approximately 3% of anticoagulated patients [17]. In these cases, continued anticoagulation therapy may lead to the resolution of LAA thrombi, with comparable success rates between NOACs and VKAs [18–20]. However, the optimal management of LAA thrombi persisting despite adequate OAC therapy has not yet been explored. Current guidelines do not provide clear recommendations; instead, they advocate for an individualized approach. According to a recently published EHRA survey, most OAC-resistant LAA thrombi are treated by switching to a different NOAC or changing to VKA [21]. In addition, the authors reported that LAA closure is often considered an option in these patients, mainly as off-label therapy, due to the lack of consistent recommendations.

## Methods

### Ethical review

The study complied with the Declaration of Helsinki and was approved by the Institutional Review Board of Semmelweis University (identifier: NNGYK/GYSZ/52316-1/2024).

### Patient selection

**Fig. 1:**
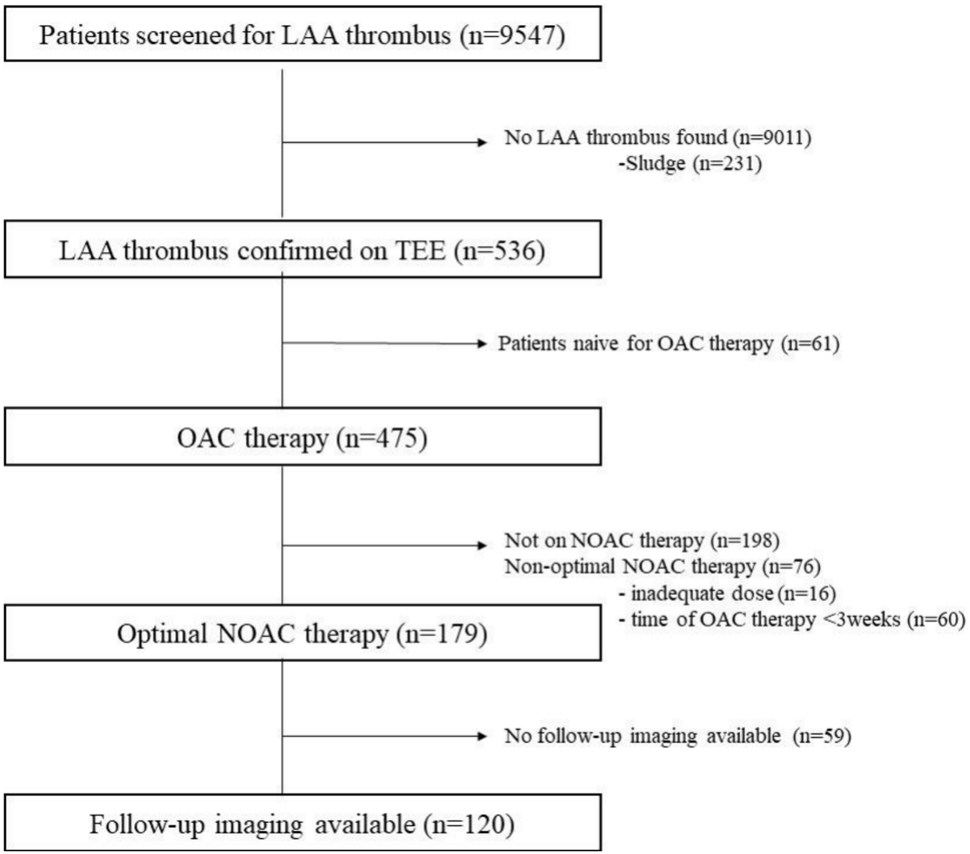
: Flowchart demonstrating the patient selection process of the study. Patients scheduled for cardioversion or catheter ablation of AF between 2014 and 2023 with LAA thrombus on transesophageal echocardiography (TEE) despite being on optimal NOAC therapy were included in the analysis. *LAA* left atrial appendage; *NOAC* Non-vitamin-K oral anticoagulant; *OAC* oral anticoagulant; *TEE* transesophageal echocardiography

In our single-center observational study, we collected the data of patients with AF, typical or atypical atrial flutter undergoing TEE before CA or ECV. Patients with a confirmed LAA thrombus were analyzed, and only patients on adequate NOAC therapy for at least 21 days before the procedure were included. Adequate dosing adjusted to age, weight, and renal function was verified in each case. Compliance was evaluated based on patient-reported adherence and the records of filled prescriptions using the National e-Health Care Cloud Hosting system. The collected data included medical history, comorbidities, and echocardiography findings (including LAA flow and left atrial parameters). The process of patient selection is demonstrated in Fig. 1.

### Transesophageal echocardiography

Transesophageal echocardiography was performed to assess the presence of LAA thrombus in patients [22], adhering to current clinical guidelines [7, 8]. Under local anesthesia with lidocaine and sedation using midazolam or propofol, the LAA was systematically evaluated using multiple imaging planes to optimize visualization and minimize artifacts. Three-dimensional multiplane imaging was employed for enhanced assessment. To exclude LAA thrombus or sludge with the highest possible accuracy, color Doppler and pulsed-wave Doppler techniques were utilized to evaluate appendage flow precisely. A positive finding was the identification of a distinct echo-dense mass within the LAA. Left atrial sludge and spontaneous echo contrast were reported but not regarded as positive findings in the present study. When deemed necessary by the primary examiner, images were independently reviewed by two experienced echocardiographers to confirm the findings. The size of the LAA thrombus was represented by its area, calculated by multiplying two measured dimensions in the imaging plane where the thrombus appeared largest.

### CT angiography

A small fraction of follow-up imaging studies were performed using CT angiography. Patients underwent contrast-enhanced computed tomography of the left atrium (LA) to rule out thrombus formation. Imaging was performed using a wide-detector cardiac CT scanner (GE CardioGraphe, GE Healthcare, Chicago, IL, USA). Patients without contraindications received 0.8 mg of sublingual nitroglycerin. Scan parameters for coronary CT angiography were tailored to the patient’s anthropometrics, with imaging completed using a rotation time of 240 ms. Systolic triggering was employed for patients with heart rates exceeding 75 beats per minute. The contrast agent Iomeprol (Iomeron 400, Bracco Ltd., Milan, Italy) was injected through an antecubital vein, using 85–95 mL at a flow rate of 4.5–5.5 mL/s in a four-phase protocol. A delayed scanning protocol was implemented to rule out false-positive findings on CT angiography caused by impaired contrast mixing due to slow flow associated with AF [23], which was shown to be a reliable alternative to TEE for detecting LAA thrombi [24]. Bolus tracking in the LA ensured appropriate scan timing [25], and images were reconstructed with statistical iterative reconstruction at level 70 [26]. The structure and Hounsfield Unit (HU) of the given area were analyzed to differentiate thrombi from contrast material defects. Thrombi appear as smooth, well-defined formations with consistently low attenuation (typically less than 50 HU in both arterial and delayed phase). Contrast-related defects due to slow flow have diffuse, irregular, or crescent-shaped appearance with higher attenuation due to contrast mixing (typically more than 100 HU and shows an increase in the delayed phase) [27].

### Classification of management strategies

The management strategy was classified into either change to a different NOAC, transition to VKA, initiation of antiplatelet therapy, or no change in antithrombotic therapy (Fig. 1).

Our endpoint was the successful LAA thrombus resolution, assessed during the follow-up imaging. This was either a repeat TEE or a left atrial CT angiography, scheduled based on the physician’s decision a minimum of 3 weeks after the index imaging. During follow-up, bleeding events were monitored and collected throughout the study period. Major bleeding was based on the Bleeding Academic Research Consortium (BARC) classification [28]. Bleeding events and their relation to antithrombotic therapy were assessed using electronic health records.

### Statistical analysis

Parameters with more than 20% missing values were excluded from our analysis. The statistical analysis was performed with the Scipy 1.8.0 library in Python (Python Software Foundation, Wilmington, DE, USA) [29, 30]. Categorical variables were presented as event numbers and percentages, while numerical variables were presented as median values and interquartile ranges (IRs). Baseline parameters were compared using the Chi-square test or Fisher’s exact test for categorical variables and the Student’s t-test (normally distributed data) or the Mann–Whitney U test (skewed data) for scalar variables. To identify predictors of LAA thrombus resolution, we performed univariable logistic regression. Statistically significant values and parameters of high clinical importance were included in a multivariable logistic regression model to identify independent predictors of the endpoint. Results were expressed with odds ratios (OR) and their corresponding 95% confidence intervals. Parameters were considered significant with a p value of less than 0.05.

## Results

**Fig. 2:**
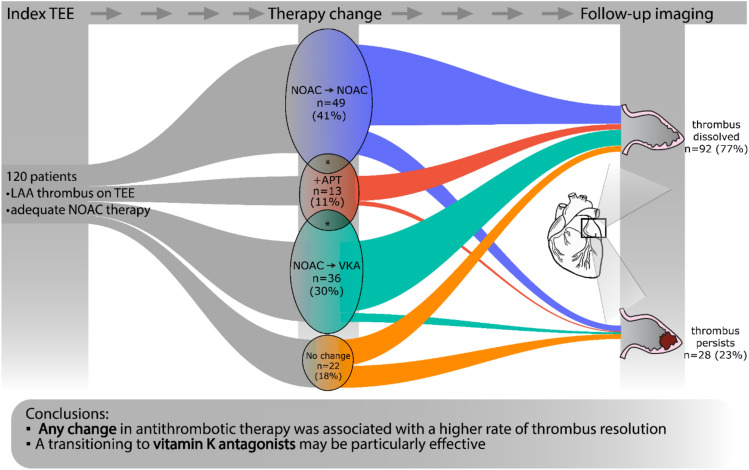
Diagram demonstrating the phases of the study. At a given time point, the width of the colored bands is proportional to the number of patients in the respective subcohort. Asterisks (*) denote the overlap between groups. Those cases where both a change in anticoagulant and augmentation with antiplatelet therapy were applied are displayed according to the change in anticoagulant. *APT* anti-platelet therapy; *NOAC* novel oral anticoagulant; *TEE* transesophageal echocardiography; *VKA* vitamin K antagonist

**Fig. 3:**
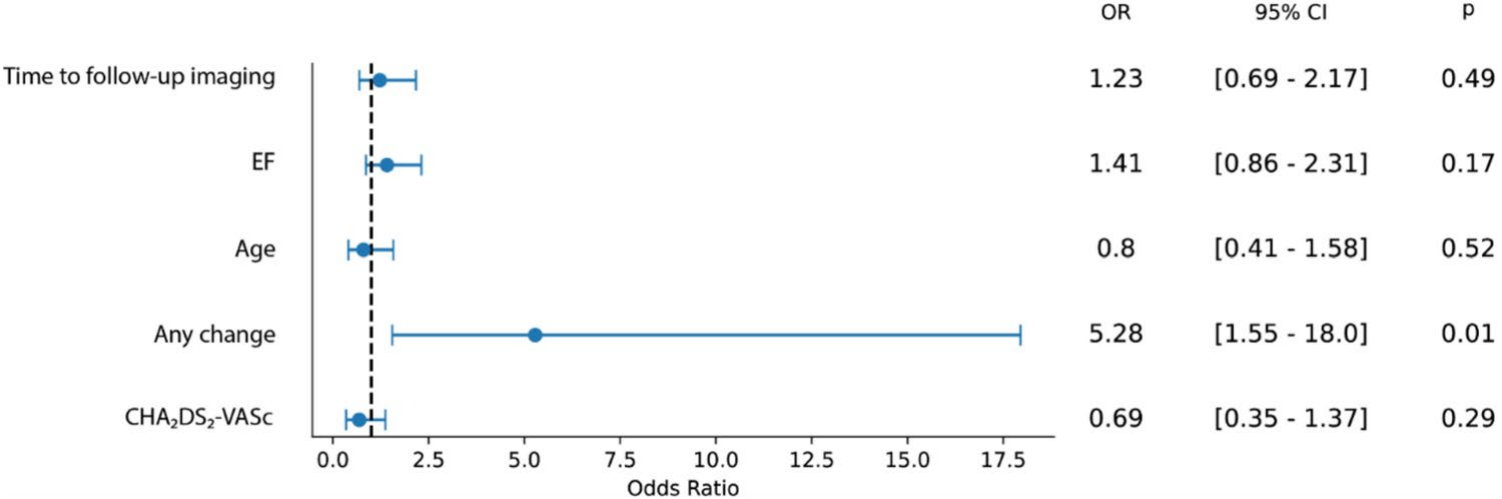
Forest plot of the multivariable logistic regression assessing the effect of any change in antithrombotic therapy, adjusted for possible confounders. *OR* odds ratio; *95% CI* 95% confidence interval. *p* < 0.05 was considered statistically significant

Out of the 9547 patients screened, we identified 536 with a solid LAA thrombus. 179 patients fit our inclusion criteria, out of which follow-up imaging was available in 120 cases. These patients had a median age of 69 (62–74) years; 77 (63%) were male. 85 (67%) patients were referred for ECV, while 35 (33%) were scheduled for CA. The most common comorbidities were hypertension (HT) in 92 (77%) and heart failure (HF) in 51 (42%) cases, while 38 patients (32%) had diabetes. The median left ventricular ejection fraction (LVEF) was 50% (40–55), and the median CHA2DS2-VASc score was 3 (2–4). The median LAA thrombus size measured on baseline TEE was 44 mm2 (29.5–71). The initial anticoagulant therapy was either rivaroxaban (*n* = 43, 36%), apixaban (*n* = 47, 39%), edoxaban (*n* = 16, 13%), or dabigatran (*n* = 14, 12%). In addition, 26 (22%) patients were on antiplatelet therapy due to coronary artery disease (CAD). Following the index TEE, the antithrombotic therapy was modified in 98 (82%) cases. A change to a different NOAC occurred in 49 (41%) cases, of which an antiplatelet agent was added in 8 (7%) cases. Switching to dabigatran was the most common choice, occurring in 34 (69%) cases, followed by apixaban (12 cases, 24%), rivaroxaban (3 cases, 6%), and edoxaban (1 case, 2%). A switch to VKA was made in 36 (30%) cases, out of which antiplatelet therapy was also initiated in 3 (2%) cases. Treatment was augmented with antiplatelet therapy alone in 13 (11%) patients. In 22 patients (18%), the initial OAC therapy remained unchanged (Fig. 2). Among those who continued their original therapy, 9 patients (41%) were on apixaban, 7 (32%) on rivaroxaban, 4 (18%) on dabigatran and 2 (9%) on edoxaban. No patients were referred for LAA occlusion procedure. Follow-up imaging was performed by TEE (*n* = 110, 92%) or CT angiography (*n* = 10, 8%) at a median of 65 (44–95) days after the index TEE. Successful thrombus resolution was achieved in 92 patients (77%). In 28 (23%) patients, a persisting LAA thrombus was found at follow-up imaging. Patients with successful thrombus resolution were younger (*n* = 67 vs. 73, *p* = 0.02), had higher median LVEF at baseline (50% vs. 42%, *p* = 0.043), and had a lower CHA2DS2-VASc score. There were no gender differences observed regarding thrombus resolution between males and females. Importantly, thrombus resolution was achieved in 80 cases (82%) in the patients with any modification in antithrombotic therapy compared to 12 (55%) of the patients who received unaltered therapy (*p* = 0.01) (Table 1). Likewise, in the univariable logistic regression analysis, any change in OAC therapy was highly predictive of thrombus resolution (OR = 3.7 [1.39–9.9], *p* = 0.01). Out of the three modification strategies (NOAC to NOAC, NOAC to VKA, and augmentation with antiplatelet therapy), a change to VKA predicted thrombus resolution (OR = 3.2 [1.02–10], *p* = 0.046). Furthermore, a higher CHA2DS2-VASc score (OR = 0.72 [0.56–0.92], p = 0.001) was also associated with less successful thrombus resolution. Diabetes (OR = 0.43 [0.18–1.03], *p* = 0.59], and lower LVEF (OR = 1.04 [1.0–1.07], *p* = 0.054) showed a trend toward persisting LAA thrombosis (Table 2). Finally, we performed multivariable logistic regression, adjusting for age, LVEF, CHA₂DS₂-VASc score, and time to follow-up imaging. We found that any change in antithrombotic therapy (OR = 5.28 [1.55–18], *p* = 0.01) was an independent predictor of thrombus resolution (Fig. 3). Switching to VKA was also assessed in the multivariate model, with a trend toward better thrombus resolution, though it did not reach statistical significance (OR = 4.02 [0.93–17.4], *p* = 0.06, Supplementary Fig. 1).

**Table 1:** Baseline characteristics comparing patients with successful thrombus resolution to those with a persisting thrombus

	All patients (*n* = 120)	Thrombus dissolved (*n* = 92)	Thrombus persisted (*n* = 28)	*p* value
*Medical history*				
Age (years)	68.7 [62.1–74.4]	66.6 [57.6–75.7]	73.8 [64.8–78.3]	**0.02**
Male sex	77 (64%)	59 (64%)	18 (64%)	0.99
Hypertension	92 (77%)	67 (73%)	25 (89%)	0.08
Diabetes	38 (32%)	25 (27%)	13 (46%)	0.05
Heart failure	51 (42%)	35 (38%)	16 (57%)	0.07
PAD	8 (7%)	6 (7%)	2 (7%)	1.00
COPD	10 (8%)	8 (9%)	2 (7%)	1.00
Persistent AF	73 (61%)	57 (62%)	16 (57%)	0.47
Stroke	12 (10%)	8 (9%)	4 (14%)	0.47
CHA2DS2-VASc	3 [2–4]	3 [2–4]	4 [2–6]	**0.01**
*Echocardiography and laboratory*				
Thrombus size (mm2)	42 [29.5–71]	42 [29.5–68]	50.6 [19.9–81.3]	0.91
LVEF (%)	50 [40–55]	50 [40–55]	42 [29–56]	**0.04**
LVEDD (mm)	52 [46–56]	52 [46–55]	52.5 [43.2–61.7]	0.67
LVESD (mm)	38 [31–44]	37 [31.5–42.5]	39.7 [29.8–49.6]	0.49
LA diameter 1 (mm)	46 [39.0–52.5]	46.0 [38.8–53.1]	45.0 [39.6–50.4]	0.45
LA diameter 2 (mm)	59 [55–63]	59 [53.5–63.5]	60 [55.3–63.5]	0.35
LAA flow (ms)	23 [19.5–29]	24 [20–30]	21 [18.2–26.8]	0.16
TAPSE (mm)	19 [16–22]	19 [17–22]	17.2 [10.9–23.6]	0.17
GFR (ml/min/1,73 m2)	66 [47.4–82.9]	67 [47.8–83.5]	63.1 [43.4–82.7]	0.69
*Management*				
Follow-up (days)	65 [44–95]	70.5 [49–112]	55.5 [34.5–90]	0.1
Any change in therapy	98 (82%)	80 (87%)	18 (64%)	**0.01**
NOAC- > VKA	36 (30%)	32 (35%)	4 (14%)	**0.04**
NOAC—> NOAC	49 (41%)	37 (40%)	12 (43%)	0.80
+ Antiplatelet	24 (20%)	20 (22%)	4 (14%)	0.39
Scheduled for ablation	34 (28%)	30 (33%)	4 (14%)	0.06

Values in bold indicate statistically significant results

*COPD chronic obstructive pulmonary disease, LA left atrial, LVEDD left ventricular end-diastolic diameter, LVEF left ventricular ejection fraction, LVESD left ventricular end-systolic diameter, NOAC novel oral anticoagulant, PAD peripheral arterial disease, TAPSE Tricuspid annular plane systolic excursion, VKA vitamin K antagonist*

**Table 2:** Univariable logistic regression showing the significant predictors of thrombus resolution

Parameters	OR (95% CI)	*p*
NOAC—> VKA	3.2 [1.02–10.0]	**0.04**
Age	0.94 [0.9–1.0]	**0.03**
Any change in therapy	3.7 [1.39–9.9]	**0.01**
LVEF	1.04 [1.0–1.07]	0.05
CHADS-Vasc Score	0.72 [0.56–0.92]	**0.01**

Values in bold indicate statistically significant results

*p* < 0.05 was considered statistically significant (in bold)

*LVEF* left ventricular ejection fraction, *NOAC* novel oral anticoagulant, *VKA* vitamin K antagonist, *OR* odds ratio, *95% CI* 95% confidence interval

In case of persisting LAA thrombus at follow-up (*n* = 28, 23%), a therapeutic decision was made out of three possible strategies: another attempt to change oral anticoagulant therapy for thrombus resolution (*n* = 13, 46%), unchanged OAC therapy with long-term rate control with either medication or AV nodal ablation (*n* = 14, 50%), or proceeding to LAA closure (*n* = 1, 4%).

Two minor bleeding events were recorded during the study period, one in the NOAC to VKA group, and the other one in the augmentation with antiplatelet therapy group. Neither of these events was defined as major bleeding or required further intervention, or discontinuation of antithrombotic therapy.

During our follow-up, no stroke or other systemic embolism was observed in either of the analyzed patients.

## Discussion

In the present study, we show an association between the modification of the antithrombotic regimen and the successful resolution of the LAA thrombus in patients who were originally on adequate NOAC therapy. Our results indicate that any change in baseline antithrombotic therapy may carry potential clinical benefit compared to no change in antithrombotic therapy. In addition, our data suggest that switching to VKA might be reasonable in this population.

Recent studies provide growing evidence for rhythm control as the superior therapeutic strategy in AF management [4, 5]. While maintaining sinus rhythm carries long-term benefits, an LAA thrombus contraindicates any rhythm control modality, making rate control the only viable option for these patients. A strategy sometimes employed in patients with a persistent thrombus is LAA closure, which was also observed in a minority of our cohort. While observational studies suggest it may be promising, LAA closure is primarily considered off-label when performed in the presence of an LAA thrombus [31]. In addition, it is still unclear whether a successful procedure warrants subsequent rhythm control strategies [32].

Current guidelines permit ECV without TEE if adequate anticoagulant therapy is maintained for at least three weeks before the procedure. This is based on an analysis of the ENSURE-AF trial, which found similar incidences of stroke with and without pre-procedural TEE in patients on adequate anticoagulation. However, this trial was not designed to assess the role of LAA imaging, and patients with a higher thromboembolic risk were more likely to undergo TEE. Interestingly, in a meta-analysis comprising 14,653 patients, Lurie et al. found an LAA-thrombus in 3% of AF patients despite optimal anticoagulation [17]. Therefore, we believe that pre-operative LAA imaging still has a role in patients with a high thromboembolic risk and that the presence of an LAA thrombus significantly contributes to the prevalence of stroke and systemic thromboembolism. Therefore, there is an unmet need for tailored antithrombotic therapy for OAC-resistant LAA thrombi to achieve rhythm control in this patient population.

Multiple studies provide evidence that both VKA and NOAC therapy can successfully resolve existing LAA thrombi [33–35]. One small randomized trial showed an initial benefit of rivaroxaban for thrombus resolution compared to warfarin, while at 12 weeks, both agents were similarly effective [34]. However, most previous studies excluded patients on optimal NOAC therapy at the time of the index TEE.

While the exact reason for anticoagulant-resistant LAA thrombosis remains unclear, we hypothesize that it may represent a specific patient population that is vastly underrepresented in the current literature.

A recently published retrospective study by Kolakowski et al. examined AF patients who developed LAA thrombus despite optimal OAC therapy [36]. They collected data from 129 patients comprising 181 treatment cycles. Management strategies were categorized into four groups: switching to a drug with a different mechanism of action, switching to a drug with a similar mechanism of action, initiating combination therapy, or maintaining the current treatment regimen without any changes. They found that any modification of the baseline OAC treatment significantly improved LAA thrombus resolution compared to no change in treatment. However, no specific modification strategy was significantly better than the others. Notably, only 74 patients (57.4%) in the studied cohort had previously received NOAC therapy. Our study follows a similar design but has some advantages. Specifically, we included only patients initially on NOAC therapy and only cases of first diagnosis of an LAA thrombus. Moreover, while our study reinforced the assumption that adjustments in antithrombotic therapy may be necessary for this population, we also observed a trend suggesting potential benefits when transitioning to VKAs.

While still lacking clear recommendations for NOAC-resistant thrombi, the latest guideline of the European Society of Cardiology addresses the question of stroke despite optimal NOAC therapy. In this population, the authors discourage the change to a different NOAC or the augmentation with antiplatelets to prevent recurrent stroke. This recommendation is based on three observational studies that show similar rates of recurrent ischemic stroke or intracranial hemorrhage in patients with a change in anticoagulant after the first event [37–43]. While our findings appear to contradict these results, we propose that directly assessing for an LAA thrombus offers a more sensitive method for evaluating the effectiveness of the chosen antithrombotic strategy. As a result, this study may highlight more nuanced differences in stroke risk associated with the anticoagulation approach used. Furthermore, given that the latest AF guidelines acknowledge the lack of data on NOAC-resistant thrombi, our findings may serve as an important first step in addressing this evidence gap.

## Limitations

Our study has limitations that need to be acknowledged. First, the single-center, retrospective, cohort study design may have compromised data availability and quality and may limit the widespread applicability of our results. Another limitation stems from the variability of the follow-up time. Though we found no correlation between the follow-up time and thrombus resolution, we adjusted for this parameter during the multivariate analysis. Furthermore, given the short follow-up period and the moderate sample size, our study is not sufficiently powered to draw conclusions about the association between stroke rates and thrombus resolution. Although LAA flow and LA diameters were recorded during TEE, left atrial volume index (LAVi) was not available, which could have added value to our findings. Finally, we acknowledge that the limited sample size contributes to the wide confidence intervals described in our results. Nevertheless, we enrolled all eligible cases from a tertiary referral center with a high number of scheduled cardioversions with protocol-mandated TEE spanning ten years; to our knowledge, this is the largest analyzed cohort of patients with a NOAC-resistant LAA thrombus who underwent repeat imaging.

## Conclusion

We found that in AF patients with an LAA thrombus despite adequate NOAC therapy, any change in antithrombotic treatment is associated with a higher probability of thrombus resolution. In addition, we observed a trend toward a higher rate of thrombus resolution with a change from NOAC to VKA. These results support the hypothesis that the antithrombotic therapy should be re-evaluated in such cases, and a change to VKAs might be considered. Once verified in prospective trials, these findings may provide important insight into the optimal management of this patient population and ultimately facilitate the implementation of rhythm control strategies in atrial fibrillation. 

## Supplementary Information

Below is the link to the electronic supplementary material.

Supplementary file1 (PDF 96 KB)

## Data Availability

The data that support the findings of this study are available from the corresponding author [K.V.N.], upon reasonable request.
