# Supplementary material for: Comparative analysis of therapeutic strategies in atrial fibrillation patients with left atrial appendage thrombus despite optimal NOAC therapy
Source: Clin Res Cardiol. 2025 May 6;115(6):937–45. doi: 10.1007/s00392-025-02665-w (PMC13161005; doi:10.1007/s00392-025-02665-w)
Supplement: Supplementary file 1 — Supplementary file1 (PDF 96 KB) [file 392_2025_2665_MOESM1_ESM.pdf]

Comparative analysis of therapeutic strategies in atrial fibrillation patients with left atrial appendage thrombus despite optimal NOAC therapy

Supplement

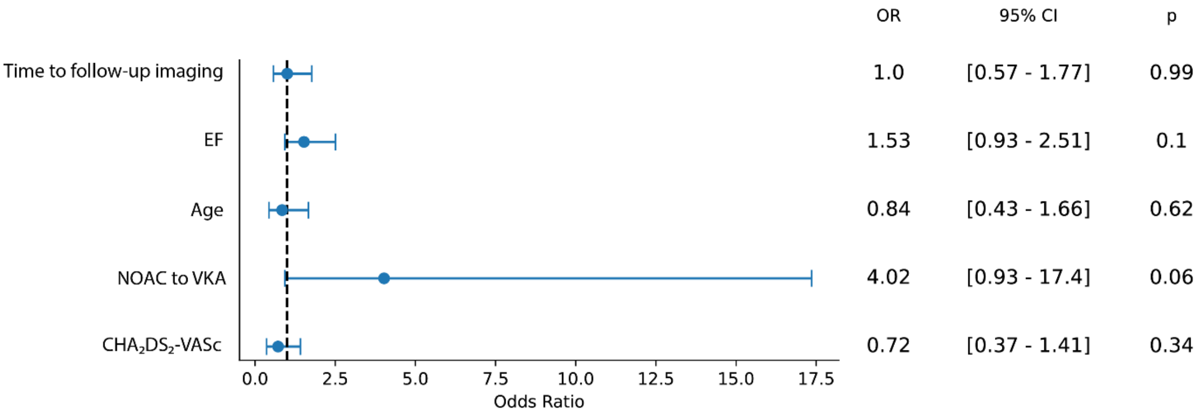

Supplementary Figure 1: Forest plot of the multivariable logistic regression assessing the change to a vitamin K antagonist, adjusted for possible confounders. OR: odds ratio; 95% CI: 95 percent confidence interval
